# Ambroxol - Resurgence of an old molecule as an anti-inflammatory agent in chronic obstructive airway diseases

**DOI:** 10.4103/0970-2113.63603

**Published:** 2010

**Authors:** P. R. Gupta

**Affiliations:** *Department of TB and Chest Diseases and Member Secretary, Medical Education Unit, SMS Medical College, Jaipur, India. E-mail: guptapr_dr@hotmail.com*

Ambroxol hydrochloride (C_13_H_18_Br_2_N_2_O) is an active N-desmethyl metabolite of bromhexine hydrochloride. Deletion of a methyl group and introduction of a hydroxyl group in a para-trans position of cyclohexyl ring have enriched ambroxol to acquire several new but important pharmacological properties, namely, surfactant stimulatory, anti-imflammatory, anti-oxidant, and local anesthetic effects in addition to the muco-kinetic and muco-ciliary effects of the parent compound. It is available in several formulations. After intravenous administration, the drug is accumulated mainly in the lungs and the elimination half life is about 10 hours. After oral administration, the drug is rapidly absorbed, bioavailability is 79% and plasma protein binding is 90%. Elimination is through a two-phase oxidative biotransformation to dibromoanthranilic acid and glucronides through cytochrome P450 3A4.[1] Recognition of the surfactant stimulatory and anti-inflammatory properties of the drug has led to resurgence of interest in the molecule in the management of difficult to treat obstructive airway disorders, where lack of surfactant and mucus stasis are bound in reciprocal causal relationship and perpetuate the inflammatory process.[2,3] Decreased steroid responsiveness has also been reported in these patients.

## EXPERIMENTAL DATA

Existing overwhelming data shows that ambroxol has surfactant stimulatory and anti-inflammatory properties. Intra-tracheal insufflation of ambroxol led to increase in bronchial secretions in animal studies. The extent of the effect was dose-dependent[4] and due to increase in secretion of surfactant[5] and mucous glycoprotein.[6] As a result the secretions become thin and less viscid. Further, in isolated lung preparations of animals, ambroxol stimulated ciliary activity and/or increased the beat frequency.[7] The drug was found to increase the volume of type II cells, thus enhancing the incorporation of labeled precursors like palmatic acid into alveolar phosphatidyl-choline and storage of lamellar bodies, thus indicating that it up regulates the production of surfactant.[8] This is also evident from the fact that pregnant animals given ambroxol during 24-26 weeks of gestation led to improved lung functions in prematurely delivered fetuses.[9] Further, treatment of animals suffering from induced acute respiratory distress syndrome with ambroxol resulted in increased survival of the animals as compared to the controls.[10] The drug up regulates surfactant production by increasing the uptake of 14C-chooline and 32P-phosphate.[11]

Experiments on neutrophils, macrophases and mast cells have shown that Ambroxol has antioxidant and anti-inflammatory properties as well. Pulmonary surfactant and inflammatory mediators share phosphatidylcholine as their substrate. By increasing production of surfactant, ambroxol reduces the production of leukotrienes, interleukin I and Tumor Necrosis Factor (TNF), the inflammatory mediators that cause inflammation and broncho-reactions.[12] In animals exposed to tobacco smoke and other toxic inhalants, ambroxol acted as free radical scavenger and was capable of protecting these animals from the oxidative stress injury.[13,14] Further, it decreased the lipopolysacharide (LPS) induced synthesis of cytokines, oxygen radicals and nitric oxide in alveolar macrophages.[15,16] Ambroxol also inhibited release of histamine and synthesis of leukotrienes from human mast cells and monocytes, the cells responsible for mediating the acute phase of immediate hypersensitivity reactions in lung, intestine and skin. Thus it reduced immediate bronchoconstriction by reducing smooth muscle contraction, vasodilatation and vascular permeability.[17]

In acute lung injury models also, the compound decreased LPS induced lung hemorrhage, edema, exudation, infiltration with neutrophills and release of cytokines.[18] Ambroxol was capable of suppressing influenza virus replication in airway fluid. Thus it improved survival rates in treated animals.[19] Ambroxol, possibly on account of increased clearance of mucus in small airways,[20] increases the penetration index for drugs in the inflamed airways.

Toxicity studies with the compound has been done in wide range of animals and has been found to be low.[20] Overdose toxicity included dyspnoea, ataxia and convulsions but subacute and chronic toxicity were distinctly uncommon and reversible in nature. It was neither embroytoxic or teratogenic, nor it affected fertility and postnatal development in rat and rabbits.[21] It was also devoid of any mutagenic or tumerogenic effect.[22]

## CLINICAL DATA

Inhaled LABA and corticosteroids form the backbone of therapy in severs obstructive airway diseases but its efficacy is sometimes limited by steroid resistance and the poor penetration of the drugs down the airways. Methylxanthines have long been recognized for their use in the treatment of obstructive airway diseases, largely due to the ability of these drugs to elicit broncho-dilatation and the newly found role of unlocking steroid resistance but frequent adverse effects have limited its role.

Ambroxol has been available in market since 1973. Since then, the drug has been administered to thousands of patients. The reported adverse reactions are low and include skin rashes, nausea, vomiting, abdominal pain and dyspepsia. Anaphylactic reactions and allergic reactions are rare. Thus the drug is considered as safe for human consumption. It has mucoactive, secretolytic, secretomotoric, anti-imflammatory and anti-oxidant actions. Thus it may be of immense value in the management of difficult-to-treat obstructive airway disorders, namely, COPD and asthma in smokers. Indeed, in several clinical studies in COPD patients, ambroxol has been shown to reduce acute exacerbations, work off days and/or hospital admissions, more particularly in those with severe disease,[23‐25] but the drug had no effect on lung functions.

Nonetheless, the reduction in acute exacerbations is also of paramount importance as it reduces cost of treatment in these patients.[26] Guidelines on management of COPD also highlight this.[27] Further, in AMETHIST study,[25] where patients were treated for 6 months, it was possible to discontinue oral corticosteroids. Guyatt *et al*.[28] reported that the drug has no effect on health related quality of life. However, this study had limitations in terms of sample size and duration.

Most clinical trials with ambroxol in COPD and asthma patients have been done in combination with methylxanthines. The combination salt, acebrophylline, is obtained by targeted salification of ambroxol base and theophylline 7 acetic acid in a stoichiometric ratio so that after absorption, high plasma levels of ambroxol are obtained along with low levels of the xanthine derivative. Pozzi[29] has reviewed the anti-inflammatory effects of the compound and has summarized that given orally, acebrophylline reduces the hyper-responsive inflammatory responses in these conditions, more particularly, the broncho-spastic episodes.

Larger and well conducted controlled trials are the need of the hour to fully assess the clinical role of ambroxol alone or as acebrophylline in the management of the difficult-to-treat obstructive airway disorders like severe COPD and severe asthma in smokers, when unlocking steroid responsiveness emerges as an important issue.

## References

[1] Jauch R, Bozler G, Hammer R (1978). Ambroxol: Studies of metabolism in man and quantitative determination in biological samples. Arzneimittelforschung Drug Res.

[2] Rensch H, Von Seefeld H, Robertson B, Van Golde LM, Batenburg JJ (1984). Surfactant-mucus interaction. Pulmonary surfactant.

[3] Bashmakov YK, Bryuscina TS (September 1989). The phospholipids of pulmonary surfactant as metabolic predecessor of SRSA. (abstr) In abstracts of the Congress of the European Academy of Allergology and Clinical Immunology.

[4] Pueschmann S, Engelhorn R (1978). Pharmacological study on the bromhexine-metabolite ambroxol. Drug Res.

[5] Miyata T, Kai H, Saito M (1986). Effects of ambroxol on pulmonary surfactant.Analysis of fatty acid composition of phosphatidylcholine in the sputum and normal respiratory tract fluid in rabbits. Fol Pharmacol Jpn.

[6] Kyle H, Widdicombe JG (1987). Secretion of mucus induced by ambroxol in the ferret trachea. Eur J Respir Dis.

[7] Iravani J, Melville GN (1974). Mucociliary function of the respiratory tract as influenced by drugs. Respiration.

[8] Elemer G, Kapanci Y (1983). Effect of ambroxol on pneumocyte Type II cell: A morphological and biochemical study. Curr Probl Clin Biochem.

[9] Lachmann B, Tischer AB, Grossmann G, Nilsson R, Robertson B (1981). Lung compliance and alveolar expansion in the artificially ventilated premature newborn rabbit after maternal treatment with ambroxol. Respiration.

[10] Dauberschmidt R, Kuckelt W, Bender V, Hieronymi U, Mrochen H, Winsel K (1980). Effects of bromhexine metabolite VIII (NA 872) in an animal model of the respiratory distress syndrome. Bull Eur Physiopathol Resp.

[11] Post M, Batenburg JJ, Schunrmans EA, Oldenbora V, Vonder Molen AJ, Ton Golde LM (1983). The perfused rat lung as a model for studies on the formation of surfactant and the effect of ambroxol on this process. Lung.

[12] Bianchi M, Mantovani A, Erroi A, Dinarello A, Ghezzi Pl (1990). Ambroxol inhibits interleukin 1 and tumor necrosis factor production in human mononuclear cells. Agent Actions.

[13] Nowak D, Antczak A, Krol M, Biolosiewicz, Pietras T (1994). Antioxidant properties of ambroxol. Free Radiat Biol Med.

[14] Lee CS, Jang YY, Song JS, Yoon YC, Hon ES (2002). Ambroxol inhibits peroxynitrite-induced damage of a α1-antiproteinase and free radical production in activated phygocytic cells. Pharmacol Toxicol.

[15] Jang YY, Song JH, Shin YK, Hans ES, Lee CS (2003). Depressant effects of ambroxol and erdosteine on cytokine synthesis, granule enzyme release, and free radical production in rat alveolar macrophages activated by lipopolysaccharide. Pharmacol Toxicol.

[16] Severina IS, Bussygina OG, Pyatakova NV, Kinopor YV, Krosrioperor RA (2000). Ambroxol as an inhibitor of nitric oxide-dependent activation of soluble guanylate cyclase. Eur J Pharmacol.

[17] Gibbs BF, Wolff HH, Grabbe J (2000). Ambroxol inhibitors IgE-dependent mediator secretion from human skin mast cells. Inflamm Res.

[18] Su X, Wang L, Song Y, Bai C (2004). Inhibition of inflammatory responses by ambroxol: A mucolyte agent, in a murine model of acute lung injury induced by lipopolysaccharide. Intensive Care Med.

[19] Yang B, Yao DF, Ohuchi M, Ide M, Yono M, Okumura Y (2002). Ambroxol suppresses influenza-virus proliferation in the mouse airway by increasing antiviral factor levels. Eur Respir J.

[20] Weiss T, Dorow P, Felix R (1981). Mucociliary clearance under the secretolytic influence of ambroxol. Prax Pneumol.

[21] Tsunenari Y, Kast A, Honma M (1981). Toxicity studies with ambroxol (Na 872) in rats, mice and rabbits. Pharmacometrics.

[22] Iida H, Kast A, Tsunenari Y (1981). Teratology studies with ambroxol (NA-872) in rats and rabbits. Pharmacometrics.

[23] Grassi V, Daniotti S, Zavattini G (1985). Ambroxol retars in the prevention of exacerbations in chronic bronchitis: Controlled double-blind study versus placebo – preliminary study. Lotta Contro Tuberc Malat Polm Soc.

[24] Alcozer G, Barattini DF, Daniotti S (1989). Prevention of chronic bronchitis exacerbations with ambroxol (Mucolsolvan Retard): An open, long-term, multicenter study in 5635 patients. Respiration.

[25] Malerba M, Ponticiello A, Radaeli A (2004). Effect of twelve-months therapy with oral ambroxol in preventing exacerbations in patients with COPD: Double-blind, randomized, multicenter, placebo-controlled study (the AMETHIST Trial). Pulm Pharmacol Ther.

[26] Hilleman DE, Dewan N, Malesker M, Friedmon M (2000). Pharmacoeconomic evaluation of COPD. Chest.

[27] Rabe KF, Hurd S, Anzueto A (2007). Global strategy for the diagnosis, management, and prevention of chronic obstructive pulmonary disease: GOLD executive summary. Am J Respir Crit Care Med.

[28] Guyatt GH, Townsend M, Kazim F, Newhouse MT (1987). A controlled trial of ambroxol in chronic bronchitis. Chest.

[29] Pozzi E (2007). Acebrophylline: An airway mucoregulator and anti-inflammatory agent. Monaldi Arch Chest Dis.

